# Cross-sectional and longitudinal associations between the 24-hour movement behaviours, including muscle and bone strengthening activity, with bone and lean mass from childhood to adolescence

**DOI:** 10.1186/s12889-024-17711-x

**Published:** 2024-01-19

**Authors:** Annie M. Skinner, Alan R. Barker, Sarah A. Moore, Sonja Soininen, Eero A. Haapala, Juuso Väistö, Kate Westgate, Soren Brage, Timo A. Lakka, Dimitris Vlachopoulos

**Affiliations:** 1 Children’s Health and Exercise Research Centre, University of Exeter, Exeter, UK; 2 Institute of Biomedicine, University of Eastern Finland, Kuopio, Finland; 3 School of Health and Human Performance, Dalhousie University, Halifax, Canada; 4 Physician and Nursing Services, Health and Social Services Centre, Wellbeing Services County of North Savo, Varkaus, Finland; 5 Faculty of Sport and Health Sciences, University of Jyväskylä, Jyväskylä, Finland; 6 MRC Epidemiology Unit, University of Cambridge, Cambridge, UK; 7 Department of Clinical Physiology and Nuclear Medicine, Kuopio University Hospital, Kuopio, Finland; 8 Foundation for Research in Health Exercise and Nutrition, Kuopio Research Institute of Exercise Medicine, Kuopio, Finland

**Keywords:** DXA, Paediatrics, Bone mineral content, Accelerometry, Moderate-to-vigorous physical activity, Sport, Exercise, Sedentary behaviour, Sleep

## Abstract

**Background:**

This study aimed to assess whether moderate-to-vigorous physical activity (MVPA), sport and exercise as a proxy measure of muscle and bone strengthening activity, sedentary behaviour, and sleep were associated with total-body-less-head (TBLH) bone mineral content (BMC) and TBLH lean mass cross-sectionally and longitudinally from age 6 to 9 years and age 9 to 11 years to age 15 to 17 years.

**Methods:**

We used longitudinal data from a population sample of Finnish children from the Physical Activity and Nutrition in Children study (age 6 to 9 years: *n* = 478, 229 females; age 9 to 11 years: *n* = 384, 197 females; age 15 to 17 years: *n* = 222, 103 females). Linear regression analysed the cross-sectional and longitudinal associations between accelerometer-assessed MVPA, sedentary time and sleep, and questionnaire-assessed sport and exercise participation and screen time with dual-energy X-ray absorptiometry-assessed TBLH BMC and lean mass.

**Results:**

In females, MVPA at age 6 to 9 years was positively associated with TBLH BMC at age 15 to 17 years (β = 0.008, *p* = 0.010). Sport and exercise at age 9 to 11 years was positively associated with TBLH BMC (β = 0.020, *p* = 0.002) and lean mass (β = 0.343, *p* = 0.040) at age 15 to 17 years. MVPA at age 9 to 11 years was positively associated with TBLH lean mass (β = 0.272, *p* = 0.004) at age 15 to 17 years. In males, sleep at age 6 to 9 years was positively associated with TBLH lean mass (β = 0.382, *p* = 0.003) at age 15 to 17 years. Sport and exercise at age 9 to 11 years was positively associated with TBLH BMC (β = 0.027, *p* = 0.012) and lean mass (β = 0.721, *p* < 0.001) at age 15 to 17 years.

**Conclusions:**

Promoting engagement in the 24-hour movement behaviours in childhood, particularly sport and exercise to strengthen muscle and bone, is important in supporting bone and lean mass development in adolescence.

**Trial registration:**

NCT01803776; first trial registration date: 04/03/2013.

## Background

Physical activity (PA) during childhood and adolescence is a well-established and important factor in determining peak bone mass, achieved by young adulthood [1–3]. Around 40% of young adult bone mineral content (BMC) is accrued in the 4 years surrounding peak height velocity [4], so understanding the association between PA and BMC during childhood and adolescence is particularly important in enhancing bone accrual, and consequentially peak bone mass. The relationship between PA and BMC is mediated by skeletal muscle, as muscle contractions transfer forces to the bones, and the skeleton adapts in response to this loading [5]. During childhood and adolescence, lean mass increases substantially [6]. There are critical synergies between the trajectories of bone and lean tissue accrual, whereby peak height velocity is observed, followed by peak lean mass velocity, and then by peak BMC velocity, and lean mass is consistently positively related to BMC in children and adolescents [6–8]. Therefore, considering muscle outcomes alongside BMC is important in understanding the relationship between PA and bone in childhood and adolescence.

There is increasing interest in how the 24-hour day is distributed across the movement continuum, between sleep, sedentary behaviour, and PA, and how each of these components may influence health outcomes [9]. This has led to the development of the Canadian 24-Hour Movement Guidelines for Children and Youth, published in 2016 [10], followed by similar guidelines from Australia [11] and the World Health Organization (WHO) [12]. The guidelines promote engaging in moderate-to-vigorous PA (MVPA), muscle and bone strengthening activities, and light PA (LPA), sufficient sleep, and limiting screen time and sitting time [9, 11], with the WHO highlighting that any increase in PA and reduction in sedentary behaviour may be beneficial for health [12]. These recommendations are to improve physical and psychosocial health across childhood, adolescence and into adulthood, and to promote healthy habits into young adulthood. This is particularly relevant as PA tends to decrease and sedentary behaviours tend to increase across puberty [13].

Moderate-to-vigorous PA (MVPA) has been positively associated with BMC in children and adolescents, with those who do more MVPA during childhood having improved muscle and bone outcomes in late adolescence and young adulthood, even after declines in MVPA during adolescence [3, 14]. However, the WHO recommendation regarding participation in MVPA is largely based on evidence on improving adiposity and other cardiometabolic risk factors and is not necessarily as relevant for muscle and bone health [12]. The importance of specific muscle and bone strengthening activities is acknowledged in the WHO guidelines, as well as national guidelines across the globe [9, 12, 15, 16]. However, aerobic activities remain the primary focus of the guidelines, though the guidelines do also describe some recommendations of muscle and bone strengthening activities [17]. This is reflected in surveillance of adherence to the PA guidelines, which tends to focus solely on the MVPA component, as highlighted by a 2020 review which found that in the UK, none of the childhood PA surveys assessed muscle and bone strengthening activity [18]. Given that it is during childhood and adolescence when bone is most responsive to PA [19], it is crucial to target muscle and bone strengthening activity in childhood and adolescence. A 2020 rapid review of evidence found that weight-bearing activities, including weight-bearing sports participation, do improve muscle and bone outcomes in children and adolescents [20]. However, as much of this evidence is based on studies in athletes, whose level of training may be far above that of the general population, are unique in terms of physique and body composition, and are likely genetically predisposed to be an athlete, further research is needed to investigate the relationship between muscle and bone strengthening activity and muscle and bone health in the general population. The current evidence is also based on intervention studies, in which the types of activities programmed may not reflect every day PA choices of children and adolescents [19]. As such, it is unclear whether participation in self-selected muscle and bone strengthening activities, such as sport and exercise, is associated with better muscle and bone outcomes in the general population, and if so, whether these associations persist from childhood into adolescence and young adulthood. This is particularly important to consider, given that organised sport and exercise account for a large part of children’s PA [21].

Investigating movement behaviours individually, such as MVPA, has its limitations as it does not account for the role of the other important movement behaviours, such as sedentary time and sleep, which can have a reverse of additional effect on musculoskeletal phenotypes [22]. Although being physically active is considered important for muscle and bone health (particularly when it is weight-bearing and dynamic in nature), PA may only be one part of the equation, and reducing sedentary behaviour and sleeping optimally may also contribute to better muscle and bone health by increasing PA levels during the day. Meanwhile, In sedentary behaviour, the musculoskeletal system in unloaded, which may have deleterious effects on muscle and bone [23]. However, a systematic review found strong evidence to suggest no association between accelerometer-measured sedentary behaviour after adjustment for MVPA and insufficient evidence for an association between self- or proxy-reported sedentary behaviour with total body bone outcomes in children and adolescents, though the authors highlight a lack of high-quality evidence with limited longitudinal studies [23]. Evidence for the relationship between sedentary behaviour and lean mass in children and adolescents is limited, and the studies that have investigated these relationships have reported conflicting findings between sedentary behaviours with fat-free mass based on skinfold measures or bioelectrical impedance [24, 25]. It has been hypothesized that sleep may also influence bone health, as bone turnover markers peak overnight [26], and sleep restriction has been shown to lead to lower levels of bone formation markers and unchanged or higher levels of bone resorption markers in adult males, which could ultimately lead to bone loss [27]. However, there is limited research into the relationships between sleep and bone health in children, and the current evidence has shown conflicting results and is limited to cross-sectional studies [28, 29]. Sleep deficit downregulates hormones important in protein synthesis, and therefore in muscle mass maintenance [30]. However, current evidence supports sleep has either no [31] or negative [32] association with fat-free mass in children. Therefore, it remains unclear whether sedentary behaviour and sleep are important for muscle and bone health in childhood and adolescence.

Although the 24-hour movement behaviours have been investigated in relation to other health outcomes, to the best of our knowledge, the associations of the 24-hour movement behaviours of MVPA, muscle and bone strengthening activity, sedentary behaviour, and sleep have yet to be investigated in relation to muscle and bone health longitudinally from childhood to adolescence. Therefore, the aim of this study was to assess whether MVPA, sport and exercise (as a proxy measure of muscle and bone strengthening activity), sedentary behaviour (i.e., sedentary time, screen time), and sleep are associated with total-body-less-head (TBLH) BMC and TBLH lean mass by dual energy X-ray absorptiometry (DXA) cross-sectionally (at age 6 to 9 years, age 9 to 11 years, age 15 to 17 years), and longitudinally (from age 6 to 9 years and age 9 to 11 years to age 15 to 17 years).

## Methods

### Study design and participants

The Physical Activity and Nutrition in Children (PANIC) study (ClinicalTrials.gov registration number NCT01803776) is an 8-year controlled lifestyle intervention study in a population sample of Finnish children that continues as a follow-up study [33]. Children aged 6 to 9 years who were registered for the first grade in one of the 16 public schools in the city of Kuopio, Finland, in 2007 to 2009 were invited to participate in baseline examinations between 2007 and 2009. Children were eligible to participate if they had no disability that could prevent their participation in the assessments or the intervention and if both the child and their parents and/or caregivers were able to communicate in Finnish to fill out the questionnaires and participate in the intervention. Of the 736 children invited to participate at baseline, 512 attended the baseline examinations at age 6 to 9 years, of which 504 were included in the final baseline sample. At age 9 to 11 years, 438 attended examinations, which took place between 2009 and 2011. At age 15 to 17 years, 277 attended examinations which took place between 2015 and 2017. Additional information about the PANIC study is presented elsewhere [34–36].

**Fig. 1:**
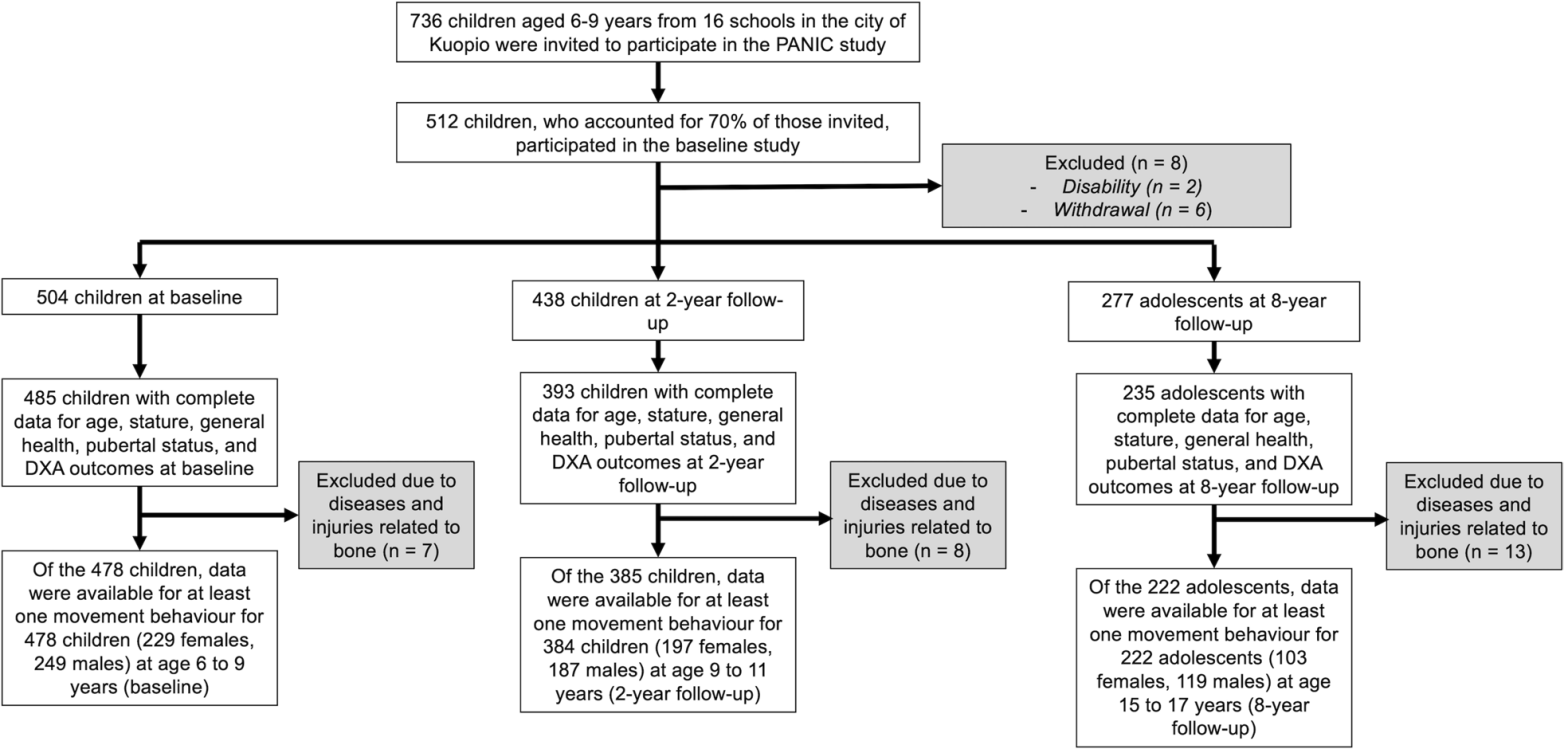
Participant flow chart. The children and adolescents included in 2-year follow-up and 8-year follow-up are part of the original baseline sample. DXA, dual energy X-ray absorptiometry; MVPA, moderate-to-vigorous physical activity; PANIC, Physical Activity and Nutrition in Children

This study used longitudinal data from baseline (at age 6 to 9 years), 2-year follow-up (at age 9 to 11 years) and 8-year follow-up (at age 15 to 17 years). For the present analyses, we excluded participants who used oral corticosteroids at any timepoint, as this could influence BMC [37], and participants with musculoskeletal injuries and diseases. Complete and valid data for age, stature, general health, pubertal status, DXA outcomes, and at least one movement behaviour were available for 478 children (229 females, 249 males) at age 6 to 9 years, 384 children (197 females, 187 males) at age 9 to 11 years, and 222 adolescents (103 females, 119 males) at age 15 to 17 years. The participants included in these analyses did not differ in age, stature, pubertal status, weight status, TBLH BMC, lean mass, or fat mass to the participants who did not have complete data. Inclusion and exclusion criteria are displayed in Fig. 1. The study protocol was approved by the Research Ethics Committee of the Hospital District of Northern Savo, and the study was conducted according to the ethical guidelines of the Declaration of Helsinki. At baseline and 2-year follow-up, the parents and/or caregivers of the children provided their written informed consent, and the children provided their assent to participation. At 8-year follow-up, the parents and/or caregivers and the adolescents gave their written informed consent.

### Assessment of general health and pubertal status

General health was assessed by a questionnaire including items on children’s and adolescent’s chronic diseases and allergies diagnosed by a physician and information on children’s and adolescent’s medication use, completed by parents and/or caregivers. Pubertal status was assessed by a research physician according to stages described by Tanner [38]. Females were defined as pubertal if their breast development had started (Tanner stage ≥ 2), and males were defined as pubertal if their testicular volume assessed by an orchidometer was ≥ 4 mL (Tanner stage ≥ 2) [38].

### Anthropometry

At all timepoints stature was measured three times to an accuracy of 0.1 cm. Body weight was measured twice using the InBody 720 bioelectrical impedance analysis device (Biospace, Seoul, South Korea) to an accuracy of 0.1 kg, with participants in a fasted state, and emptied bladder. For stature and body weight, the mean of the values was used in analyses. Body mass index (BMI) (kg/m^2^) was calculated, BMI standard deviation score (SDS) was calculated using the Finnish reference values, and the BMI cut-offs were applied to classify children and adolescents as thin, normal weight, or living with overweight or obesity as it related to their weight status [39, 40].

### Assessment of bone mineral content and body composition

TBLH BMC (kg), areal bone mineral density (aBMD) (g/cm^2^), lean mass (kg), and fat mass (kg) were measured by a research nurse using the Lunar Prodigy Advance DXA device (GE Medical Systems, Madison, WI, USA) and the Encore software, Version 10.51.006 (GE Company, Madison, WI, USA). TBLH BMC was used as the bone outcome of interest, as this is more appropriate than TBLH aBMD in longitudinal studies of bone accrual [4, 41, 42]. TBLH lean mass was considered as a secondary outcome, as lean mass is a key determinant of BMC, and the relationship between PA and BMC is mediated by lean mass [14]. DXA provides valid and reliable data on BMC and body composition in children and adolescents (coefficient of variation = 0.01 – 4.37%) [43, 44].

### Assessment of 24-hour movement behaviours

MVPA, muscle and bone strengthening activity, sedentary behaviour, and sleep were assessed, as these behaviours are outlined in the Canadian 24-hour Movement Guidelines for Children and Youth [10], the Australian 24-hour Movement Guidelines for Children and Young People [11] and, with the exception of sleep, the WHO Guidelines on Physical Activity and Sedentary Behaviour for Children and Adolescents [12].

#### Moderate to vigorous physical activity

PA was assessed using Actiheart (CamNtech Ltd, Papworth, UK), a combined heart rate and movement sensor [45, 46], using methods described previously in this cohort [35, 47, 48]. Heart rate and acceleration were recorded in 60-second epochs. At age 6 to 9 years and age 9 to 11 years, the children were asked to wear the monitor continuously for a minimum of two weekdays and two weekend days, including during sleep and water-based activities. At age 15 to 17 years, adolescents were requested to wear the monitor continuously for seven days, including sleep. Heart rate data were cleaned [49] and individually calibrated using parameters obtained from cycle ergometer tests where available [50] and were combined with acceleration data to derive PA energy expenditure. PA intensity was modelled from the combined sensing signal using a branched equation framework [47, 51, 52]. MVPA was categorised as time spent > 4 metabolic equivalents, with 3.5 mL VO_2_/min/kg used to define resting metabolic rate [53]. Non-wear time was classified as zero-acceleration lasting > 90 minutes combined with non-physiological heart rate [47]. Diurnal imbalance in non-wear was minimised when summarising the data to reduce bias and error as previously described [47, 54]. A valid PA measurement was defined as ≥ 48 hours of good-quality data with ≥ 32 hours of weekday data and ≥ 16 hours of weekend data as well as ≥ 12 hours of morning, noon, afternoon, and evening wear time to protect against bias from over-representation from specific times of day and to optimise the diurnal bias minimisation procedure. The children and adolescents were defined as meeting the PA guidelines if they had at least an average of 60 minutes of MVPA per day, as described in the WHO guidelines [12].

#### Muscle and bone strengthening activity

At age 6 to 9 years and 9 to 11 years, sport and organised exercise participation were assessed with the PANIC Physical Activity Questionnaire, which was filled out by parents or caregivers with their child [55]. The questionnaire asked how many times a week the child participated in sports or organised exercise, and the average duration of the sessions, expressed in hours per session to an accuracy of half an hour, during a usual week [55, 56]. At age 15 to 17 years, sport and organised exercise participation were assessed with the PANIC Physical Activity Questionnaire for Adolescents and Adults, a retrospective questionnaire over the prior 12 months to account for seasonal variation in activities [57]. Adolescents were provided a list of 42 physical activities and asked whether they had participated in any of the activities, in how many months of the prior year, how many times per week, and how many hours or minutes per session.

Sports and organised exercise participation was used as a proxy measure for muscle and bone strengthening activity. Previous studies which have assessed adherence to the muscle and bone strengthening guidelines have used frequency questionnaires, with activities such as push-ups, sit-ups and weight-lifting classed as muscle strengthening, and a wide variety of activities classed as bone strengthening, including basketball, cheerleading, dance, hockey, gymnastics, soccer, racket sports, and weight lifting [58]. In addition, the Finnish Physical Activity Guidelines for children and adolescents suggest body weight training, gym training, group PA, and stair walking as muscle strengthening activity, and gymnastics, athletics and ball games as bone strengthening activity examples [59]. The types of activities classed as muscle and bone strengthening therefore tend to be captured within ‘sport and organised exercise participation’. However, there is no recommended session duration defined in the muscle and bone strengthening activity guidelines [12]. Hence, children and adolescents who participated in sport and organised exercise at least three times a week were defined as meeting the guidelines [12].

#### Sedentary behaviour

As limiting total sedentary time and recreational screen time is recommended by the WHO [12], measures of both sedentary time and screen time were included in this study. Sedentary time was modelled from the combined heart rate and movement sensor data as described above, and sedentary time was categorised as time spent ≤ 1.5 metabolic equivalents, with 3.5 mL VO_2_/min/kg used to define resting metabolic rate [12, 52]. To further describe each child’s sedentary behaviours, screen time was assessed with the PANIC Physical Activity Questionnaire, which was filled out by the parent or caregivers with their child [60]. The questionnaire asked for time spent watching television and videos, using computers and playing video and console games, and using mobile phones and playing mobile phone games [60]. Although self-report and proxy-report measures of screen time have mixed validity and reliability in the literature, the lack of gold standard method for assessing screen time makes it difficult to accurately assess validity and reliability, and self- and proxy-report measures are commonly used in research [61]. The children and adolescents were defined as meeting the guidelines if their average daily screen time was no more than 2 hours [10, 62].

#### Sleep

Sleep duration was assessed from the combined heart rate and movement sensor data by a trained exercise specialist and confirmed by an experienced researcher [47]. The time of falling asleep was defined as a time point when accelerometer counts were zero with a heart rate plateau, and wake time was defined by an increase in accelerometer counts from zero and an increase in heart rate above plateau level. The children at age 6 to 9 years and age 9 to 11 years were defined as meeting the guidelines if their average daily sleep was between 9 and 11 hours, and the adolescents at age 15 to 17 years if their average daily sleep was between 8 and 10 hours [10, 60, 62, 63].

### Statistical analysis

Analyses were performed with Stata/SE for Mac software, Version 17.0 (StataCorp LLC, College Station, TX, USA). There were no differences between the children who did meet the inclusion criteria and those who did not meet the inclusion criteria in terms of age, stature, BMI categories, and pubertal status at age 6 to 9 years, age 9 to 11 years, and age 15 to 17 years. Further, the adolescents at age 15 to 17 years who met the inclusion criteria for the longitudinal analysis did not differ in terms of age, stature, BMI categories and pubertal status at age 6 to 9 years, age 9 to 11 years and age 15 to 17 years from those who did not. Therefore, we proceeded with a complete-case analysis.

The means and standard deviations (SDs) or the medians and interquartile ranges (IQRs) were calculated stratified by sex, for each timepoint. We stratified all further analyses by sex, based on the biological differences in stature, TBLH BMC, lean mass and fat mass between females and males in the studied age group [6, 64, 65].

Cross-sectional analyses were carried out at age 6 to 9 years, age 9 to 11 years, and age 15 to 17 years. Linear regression was used to assess the associations of minutes per day of MVPA, sport and exercise, sedentary time, screen time, and sleep with TBLH BMC and TBLH lean mass. Analyses were adjusted for age, stature, pubertal status, study group (control or intervention), and TBLH fat mass. When considering TBLH BMC as the outcome, we adjusted additionally for lean mass. Further, estimates for MVPA and sedentary time were mutually adjusted for each other, as correlation analyses for females and males separately at age 6 to 9 years, age 9 to 11 years, and age 15 to 17 years showed moderate to strong inverse relationships between these two variables (age 6 to 9 years, females: *r* = -0.58, *p* < 0.001, males: *r* = -0.59, *p* < 0.001; age 9 to 11 years, females: *r* = -0.60, *p* < 0.001, males: *r* = -0.72, *p* < 0.001; age 15 to 17 years, females: *r* = -0.63, *p* < 0.001, males: -0.66, *p* < 0.001).

Longitudinal analyses were carried out between age 6 to 9 years and age 15 to 17 years, and between age 9 to 11 years and age 15 to 17 years, as it is possible that the longitudinal associations may differ between these age groups. Linear regression was used to assess the associations of minutes per day of MVPA, sport and exercise, sedentary time, screen time, and sleep at age 6 to 9 years with TBLH BMC and TBLH lean mass at age 15 to 17 years. For all longitudinal analyses, models included adjustment for age, stature, pubertal status, study group, and TBLH fat mass at age 15 to 17 years, with additional adjustment for TBLH lean mass at age 15 to 17 years and TBLH BMC at age 6 to 9 years when considering TBLH BMC as the outcome, and with TBLH lean mass at age 6 to 9 years when considering TBLH lean mass as the outcome. As with the cross-sectional analyses, MVPA and sedentary time at age 6 to 9 years were mutually adjusted for each other. The movement behaviour of interest at age 15 to 17 years was not adjusted for, as this greatly reduced the sample size for the device-measured variables of MVPA, sedentary time, and sleep (i.e., when analysing the relationship between MVPA at age 6 to 9 years with TBLH BMC at age 15 to 17 years, MVPA at age 15 to 17 years was not adjusted for). All analyses were repeated with data from age 9 to 11 years instead of age 6 to 9 years. For both the cross-sectional and longitudinal analysis, models were computed with robust standard errors and results were expressed as regression coefficients (β) representing the changes in the outcomes per 10-minute change in the movement behaviour, their 95% confidence intervals (CI), and *p*-values.

Further longitudinal analyses were conducted by grouping the participants based on their activity status at age 6 to 9 years and age 15 to 17 years. This was only done for sport and exercise participation, as the sample size of participants with valid measurements at age 6 to 9 years and age 15 to 17 years was greatly limited for the device-measured variables (MVPA, sedentary time, sleep). A median split of average time spent in sport and exercise per day was used to categorise the participants. Participants were categorised as 1) persistently inactive (below median at age 6 to 9 years and age 9 to 11 years), 2) decreasingly active (above median at age 9 to 11 years and below median at age 15 to 17 years), 3) increasingly active (below median at age 6 to 9 years and above median at age 15 to 17 years), and 4) persistently active (above median at age 6 to 9 years and age 15 to 17 years).

Linear regression with robust standard errors was used to assess differences in TBLH BMC and lean mass at age 15 to 17 years between the persistently inactive, decreasingly active, increasingly active, and persistently active groups, adjusted for age, stature, pubertal status, study group, and fat mass at age 15 to 17 years, with additional adjustment for TBLH lean mass at age 15 to 17 years and TBLH BMC at age 6 to 9 years when considering TBLH BMC as the outcome, and with TBLH lean mass at age 6 to 9 years when considering TBLH lean mass as the outcome. Estimated marginal means for each group were computed and pairwise comparisons between each pairing were performed. All analyses were then repeated, replacing the age 6 to 9 years data with the age 9 to 11 years data. For all analyses, statistical significance was set at alpha level 0.05.

## Results

### Descriptive statistics

At age 6 to 9 years, males were taller, and had greater TBLH BMC and lean mass, and lower fat mass than females (Table 1). Males had greater MVPA, greater sport and exercise participation, no difference in sedentary time, greater screen time, and no difference in sleep duration compared to females (Table 2). A greater proportion of males met the guidelines for MVPA and muscle and bone strengthening activity than females, a greater proportion of females met the guidelines for screen time, and there was no difference in the proportion of females and males meeting the sleep guidelines (Table 2).

**Table 1:** Characteristics of females and males at age 6-9, at age 9-11, and age 15-17 years

	**Age 6 to 9 years**			**Age 9 to 11 years**			**Age 15 to 17 years**		
	**Females**	**Males**	***p* value for group difference**	**Females**	**Males**	***p* value for group difference**	**Females**	**Males**	***p* value for group difference**
	**Mean/Median (SD/IQR)**	**Mean/Median (SD/IQR)**		**Mean/Median (SD/IQR)**	**Mean/Median (SD/IQR)**		**Mean/Median (SD/IQR)**	**Mean/Median (SD/IQR)**	
Age (years)	7.62 (0.38)	7.66 (0.4)	0.24	9.73 (0.43)	9.80 (0.43)	0.079	**15.73 (0.40)**	**15.85 (0.45)**	**0.036**
Stature (cm)	**127.88 (5.65)**	**129.66 (5.6)**	**0.001**	140.06 (6.44)	141.32 (6.1)	0.051	**166.18 (5.61)**	**176.51 (7.39)**	**<0.001**
Weight (kg)	26.54 (5.08)	27.32 (5.01)	0.089	33.85 (7.36)	35.23 (7.49)	0.070	**57.79 (8.72)**	**65.13 (13.23)**	**<0.001**
BMI-SDS	-0.17 (1.05)	-0.18 (1.1)	0.94	-0.14 (1.01)	-0.09 (1.1)	0.64	0.02 (0.85)	-0.16 (1.07)	0.18
Pubertal Status									
% (cases) Tanner stage 1	96.5 (221)	98.8 (246)	0.13	**65.0 (128)**	**86.6 (162)**	**<0.001**	**0 (0)**	**0 (0)**	**0.003**
% (cases) Tanner stage 2	3.5 (8)	1.2 (3)		**35.0 (69)**	**13.4 (25)**		**0 (0)**	**0 (0)**	
% (cases) Tanner stage 3	0 (0)	0 (0)		**0 (0)**	**0 (0)**		**3.9 (4)**	**13.5 (16)**	
% (cases) Tanner stage 4	0 (0)	0 (0)		**0 (0)**	**0 (0)**		**53.4 (55)**	**61.3 (73)**	
% (cases) Tanner stage 5	0 (0)	0 (0)		**0 (0)**	**0 (0)**		**42.7 (44)**	**25.2 (30)**	
IOTF Definition									
% (cases) thin	9.2 (21)	10.8 (27)	0.50	9.6 (19)	10.2 (19)	0.81	6.8 (7)	11.8 (14)	0.29
% (cases) normal weight	76 (174)	77.1 (192)		74.6 (147)	70.6 (132)		82.5 (85)	75.6 (90)	
% (cases) overweight	10.9 (25)	7.2 (18)		12.7 (25)	15 (28)		6.8 (7)	10.9 (13)	
% (cases) obese	3.9 (9)	4.8 (12)		3 (6)	4.3 (8)		3.9 (4)	1.7 (2)	
TBLH BMC (kg)	**0.66 (0.14)**	**0.69 (0.14)**	**0.010**	**0.91 (0.21)**	**0.97 (0.21)**	**0.011**	**1.91 (0.27)**	**2.27 (0.45)**	**<0.001**
TBLH lean mass (kg)	**16.87 (1.99)**	**18.69 (2.13)**	**<0.001**	**20.83 (2.79)**	**22.93 (2.80)**	**<0.001**	**35.13 (3.83)**	**47.58 (6.82)**	**<0.001**
TBLH fat mass (kg)	**4.90 (3.67 to 7.25)**	**3.56 (2.38 to 5.85)**	**<0.001**	**7 (5.06 to 11.18)**	**6.48 (3.77 to 10.85)**	**0.014**	**15.55 (12.14 to 17.99)**	**8.18 (5.85 to 13.67)**	**<0.001**

For continuous variables, values are mean/median and standard deviation/interquartile range, and *p*-values from independent samples t-tests or Mann-Whitney U tests to test for the sex difference. For categorical variables, values are % (n), with Fisher’s exact *p*-values to test for the sex difference.

At age 6 to 9 years, *n* = 478 (229 females, 249 males). At age 9 to 11 years, *n* = 384 (197 females, 187 males). At age 15 to 17 years, *n* = 222 (103 females, 119 males).

*BMC* Bone mineral content, *BMI* Body mass index, *IOTF* International Obesity Task Force, *IQR* Interquartile range, *SD* Standard deviation, *SDS* Standard deviation score, *TBLH* Total body less head

**Table 2:** Movement behaviours of females and males at age 6-9, at age 9-11, and age 15-17 years

	**Age 6 to 9 years**			**Age 9 to 11 years**			**Age 15 to 17 years**		
	**Females**	**Males**	***p* value for group difference**	**Females**	**Males**	***p* value for group difference**	**Females**	**Males**	***p* value for group difference**
	**Mean/Median (SD/IQR)**	**Mean/Median (SD/IQR)**		**Mean/Median (SD/IQR)**	**Mean/Median (SD/IQR)**		**Mean/Median (SD/IQR)**	**Mean/Median (SD/IQR)**	
MVPA (min/day)	**85 (55 to 127)**	**126 (81 to 178)**	**<0.001**	**72 (51 to 99)**	**115 (79 to 162)**	**<0.001**	**32 (18 to 48)**	**47 (26 to 71)**	**0.033**
MVPA guidelines									
% (cases) < 60 mins/day	**28.3 (52)**	**14.8 (27)**	**0.002**	**34.4 (54)**	**17.6 (23)**	**0.001**	80.0 (32)	65.2 (43)	0.13
% (cases) ≥ 60 mins/day	**71.7 (132)**	**85.2 (155)**		**65.6 (103)**	**82.4 (108)**		20.0 (8)	34.8 (23)	
Sport and exercise participation (min/day)	**9 (0 to 17)**	**13 (9 to 21)**	**0.008**	**9 (0 to 26)**	**17 (9 to 34)**	**0.028**	14 (0 to 40)	18 (0 to 68)	0.37
Muscle and bone strengthening guidelines									
% (cases) < 3 times/week	**87.8 (201)**	**79.4 (197)**	**0.019**	76.5 (150)	67.9 (127)	0.068	70.9 (73)	61.3 (73)	0.16
% (cases) ≥ 3 times/week	**12.2 (28)**	**20.6 (51)**		23.5 (46)	32.1 (60)		29.1 (30)	38.7 (46)	
Sedentary time (min/day)	242 (131)	232 (129)	0.47	393 (108)	386 (100)	0.54	598 (143)	587 (140)	0.72
Screen time (min/day)	**92 (49)**	**113 (55)**	**<0.001**	**108 (53)**	**135 (59)**	**<0.001**	**308 (166)**	**367 (199)**	**0.017**
Screen time guidelines									
% (cases) > 2 hours/day	**20.5 (47)**	**35.9 (89)**	**< 0.001**	**30.6 (60)**	**53.5 (100)**	**< 0.001**	95.1 (98)	96.6 (114)	0.74
% (cases) ≤ 2 hours/day	**79.5 (182)**	**64.1 (159)**		**69.4 (136)**	**46.5 (87)**		4.9 (5)	3.4 (4)	
Sleep (hour/night)	9.69 (0.46)	9.65 (0.55)	0.46	**9.23 (0.55)**	**9.08 (0.48)**	**0.013**	**7.73 (0.75)**	**7.46 (0.64)**	**0.012**
Sleep guidelines									
% (cases) not meeting age-specific guidelines	9.1 (17)	11.6 (22)	0.50	31 (49)	40.5 (53)	0.108	**65.4 (53)**	**81.3 (78)**	**0.025**
% (cases) meeting age-specific guidelines	90.9 (169)	88.4 (168)		69 (109)	59.5 (78)		**34.6 (28)**	**18.7 (18)**	

*IQR* Interquartile range, *MVPA* Moderate to vigorous physical activity, *SD* Standard deviation

For continuous variables, values are mean/median and standard deviation/interquartile range, and *p*-values from independent samples t-tests or Mann-Whitney U tests to test for the sex difference. For categorical variables, values are % (n), with Fisher’s exact *p*-values to test for the sex difference

At age 6 to 9 years, for MVPA, *n* = 366 (184 females, 182 males). For sport and exercise and screen time, *n* = 477 (229 females, 248 males). For sedentary time, *n* = 365 (184 females, 181 males). For sleep, *n* = 376 (186 females, 190 males)

At age 9 to 11 years, for MVPA, *n* = 288 (157 females, 131 males). For sport and exercise and screen time, *n* = 383 (196 females, 187 males). For sedentary time, *n* = 287 (157 females, 130 males). For sleep, *n* = 289 (158 females, 131 males)

At age 15 to 17 years, for MVPA and sedentary time, *n* = 106 (40 females, 66 males). For sport and exercise, *n* = 222 (103 females, 119 males). For screen time, *n* = 221 (103 females, 118 males). For sleep, *n* = 177 (81 females, 96 males)

Meeting the MVPA guidelines was defined as at least 60-minutes average daily MVPA. Meeting the muscle and bone strengthening guidelines was defined as participating in organised sport and exercise at least 3 times per week. Meeting the screen time guidelines was defined as an average of no more than 2 hours of recreational screen time per day. Meeting the sleep guidelines was defined as achieving between 9- and 11-hours average per night for children aged 6 to 11, and as achieving between 8- and 10-hours average per night for adolescents aged 15 to 17 years

At age 9 to 11 years, males had greater TBLH BMC and lean mass, and lower fat mass than females. A greater proportion of females were pubertal compared to males (Table 1). Males had greater MVPA, greater sport and exercise participation, no difference in sedentary time, greater screen time, and less sleep compared to females (Table 2). A greater proportion of males met the guidelines for MVPA, there were no differences in the proportion of females and males meeting the muscle and bone strengthening guidelines, a greater proportion of females met the guidelines for screen time, and there were no differences in the proportion of females and males meeting the sleep guidelines.

At age 15 to 17 years, males were taller, heavier, and slightly older than females, with greater TBLH BMC and lean mass, and lower fat mass than females (Table 1). More females had achieved Tanner stage 5 compared to males. Males had greater MVPA, no difference in sport and exercise participation, no difference in sedentary time, greater screen time and less sleep compared to females (Table 2). There were no differences in the proportion of females and males meeting the MVPA guidelines, the muscle and bone strengthening guidelines, and the screen time guidelines, and a greater proportion of females met the sleep guidelines compared to males.

### Cross-sectional associations of movement behaviours with TBLH BMC and lean mass in females

At age 6 to 9 years, sedentary time was negatively associated with TBLH lean mass (Table 3). There were no other associations of the movement behaviours with TBLH BMC or TBLH lean mass at age 6 to 9 years (Table 3). At age 9 to 11 years, sport and exercise participation was positively associated with TBLH BMC (Table 3). MVPA was positively associated with TBLH lean mass, and sleep was negatively associated with TBLH lean mass at age 9 to 11 years (Table 3). At age 15 to 17 years, sleep was positively associated with TBLH BMC (Table 3). MVPA and sport and exercise participation were positively associated with TBLH lean mass at age 15 to 17 years (Table 3).

**Table 3:** Cross-sectional associations of movement behaviours with TBLH BMC and lean mass in females and males

	Age 6 to 9 years^*a*^		Age 9 to 11 years^*b*^		Age 15 to 17 years^*c*^	
	β (95% CI)	p	β (95% CI)	p	β (95% CI)	p
**Females**						
TBLH BMC						
MVPA	0.001 (-0.001 to 0.002)	0.331	0.001 (-0.003 to 0.005)	0.629	0.005 (-0.016 to 0.025)	0.652
Sport and exercise	0.003 (-0.002 to 0.008)	0.270	**0.008 (0.003 to 0.013)**	**0.004**	0.008 (-0.001 to 0.018)	0.081
Sedentary time	-0.000 (-0.001 to 0.000)	0.218	-0.001 (-0.002 to 0.001)	0.219	-0.002 (-0.007 to 0.002)	0.287
Screen time	0.000 (-0.001 to 0.001)	0.929	-0.001 (-0.003 to 0.001)	0.167	-0.001 (-0.003 to 0.001)	0.460
Sleep	0.001 (-0.002 to 0.003)	0.653	0.001 (-0.003 to 0.005)	0.794	**0.008 (0.000 to 0.015)**	**0.043**
TBLH lean mass						
MVPA	0.004 (-0.036 to 0.043)	0.851	**0.142 (0.081 to 0.204)**	**<0.001**	**0.430 (0.115 to 0.744)**	**0.009**
Sport and exercise	0.013 (-0.099 to 0.125)	0.817	0.101 (-0.020 to 0.223)	0.101	**0.358 (0.247 to 0.470)**	**<0.001**
Sedentary time	**-0.017 (-0.033 to -0.001)**	**0.033**	0.013 (-0.015 to 0.041)	0.344	-0.005 (-0.084 to 0.074)	0.895
Screen time	0.019 (-0.008 to 0.045)	0.163	0.030 (-0.009 to 0.068)	0.127	-0.008 (-0.045 to 0.029)	0.667
Sleep	0.018 (-0.046 to 0.081)	0.582	**-0.108 (-0.173 to -0.043)**	**0.001**	-0.096 (-0.218 to 0.026)	0.122
**Males**						
TBLH BMC						
MVPA	0.001 (-0.001 to 0.002)	0.233	0.000 (-0.004 to 0.004)	0.985	-0.008 (-0.024 to 0.009)	0.357
Sport and exercise	**0.004 (0.001 to 0.007)**	**0.016**	0.005 (-0.001 to 0.011)	0.100	0.011 (-0.001 to 0.022)	0.066
Sedentary time	0.000 (-0.000 to 0.001)	0.264	-0.002 (-0.003 to 0.000)	0.093	-0.003 (-0.008 to 0.001)	0.146
Screen time	-0.000 (-0.001 to 0.001)	0.984	-0.001 (-0.003 to 0.001)	0.338	**-0.002 (-0.004 to -0.000)**	**0.020**
Sleep	0.001 (-0.001 to 0.003)	0.292	0.001 (-0.003 to 0.006)	0.532	-0.005 (-0.018 to 0.008)	0.430
TBLH lean mass						
MVPA	0.035 (-0.003 to 0.073)	0.070	**0.116 (0.053 to 0.178)**	**<0.001**	0.126 (-0.240 to 0.491)	0.494
Sport and exercise	**0.091 (0.022 to 0.161)**	**0.011**	**0.221 (0.092 to 0.350)**	**0.001**	**0.337 (0.142 to 0.532)**	**0.001**
Sedentary time	**0.017 (0.000 to 0.034)**	**0.045**	**0.037 (0.006 to 0.069)**	**0.020**	-0.017 (-0.113 to 0.079)	0.726
Screen time	-0.008 (-0.034 to 0.018)	0.544	-0.037 (-0.080 to 0.006)	0.094	-0.037 (-0.080 to 0.006)	0.092
Sleep	0.028 (-0.023 to 0.078)	0.278	-0.003 (-0.098 to 0.092)	0.945	0.104 (-0.106 to 0.314)	0.328

*BMC* Bone mineral content, *MVPA* Moderate to vigorous physical activity, *TBLH* Total body less head

Values are unstandardised regression coefficients, their 95% confidence intervals, and *p*-values. The regression coefficient represents the difference in TBLH BMC, and lean mass associated with a 10-minute average daily difference in each movement behaviour

Bold emphasis indicates statistical significance (*p* < 0.05)

^a^Estimates adjusted for age, stature, pubertal status, and fat mass, measured at age 6 to 9 years, and study group. Estimates for TBLH BMC additionally adjusted for lean mass measured at age 6 to 9 years. MVPA and sedentary time are mutually adjusted for each other

^b^Estimates adjusted for age, stature, pubertal status, and fat mass, measured at age 9 to 11 years, and study group. Estimates for TBLH BMC additionally adjusted for lean mass measured at age 9 to 11 years. MVPA and sedentary time are mutually adjusted for each other

^c^Estimates adjusted for age, stature, pubertal status, and fat mass, measured at age 15 to 17 years, and study group. Estimates for TBLH BMC additionally adjusted for lean mass measured at age 15 to 17 years. MVPA and sedentary time are mutually adjusted for each other

At age 6 to 9 years, *n* = 184 females and 181 males for MVPA and sedentary time, *n* = 229 females and 248 males for sport and exercise participation, *n* = 229 females and 248 males for screen time, *n* = 186 females and 190 males for sleep

At age 9 to 11 years, *n* = 157 females and 130 males for MVPA and sedentary time, *n* = 196 females and 187 males for sport and exercise participation, *n* = 196 females and 187 males for screen time, n = 158 females and 131 males for sleep

At age 15 to 17 years, *n* = 40 females and 66 males for MVPA and sedentary time, *n* = 103 females and 119 males for sport and exercise participation, *n* = 103 females and 118 males for screen time, *n* = 81 females and 96 males for sleep

### Cross-sectional associations of movement behaviours with TBLH BMC and lean mass in males

At age 6 to 9 years, sport and exercise participation was positively associated with TBLH BMC and lean mass and sedentary time was positively associated with TBLH lean mass (Table 3). There were no other associations between the movement behaviours with TBLH BMC or TBLH lean mass at age 6 to 9 years. At age 9 to 11 years, MVPA, sport and exercise participation, and sedentary time were positively associated with TBLH lean mass. At age 15 to 17 years, screen time was negatively associated with TBLH BMC, and sport and exercise participation was positively associated with TBLH lean mass (Table 3).

### Longitudinal associations of movement behaviours with TBLH BMC and lean mass in females

MVPA at age 6 to 9 years was positively associated with TBLH BMC at age 15 to 17 years (Table 4). Sport and exercise participation at age 9 to 11 years was positively associated with TBLH BMC and lean mass at age 15 to 17 years (Table 4). MVPA at age 9 to 11 years was positively associated with TBLH lean mass at age 15 to 17 years (Table 4).

**Table 4:** Longitudinal associations of movement behaviours with TBLH BMC and lean mass in females and males

	Age 6 to 9 years ➔ Age 15 to 17 years^*a*^		Age 9 to 11 years ➔ Age 15 to 17 years^*b*^	
	β (95% CI)	p	β (95% CI)	p
**Females**				
TBLH BMC				
MVPA	**0.008 (0.002 to 0.014)**	**0.010**	0.010 (-0.001 to 0.021)	0.082
Sport and exercise	0.014 (-0.006 to 0.034)	0.160	**0.020 (0.008 to 0.032)**	**0.002**
Sedentary time	-0.000 (-0.003 to 0.002)	0.914	0.001 (-0.003 to 0.005)	0.598
Screen time	-0.001 (-0.006 to 0.004)	0.755	-0.003 (-0.009 to 0.003)	0.346
Sleep	-0.003 (-0.015 to 0.008)	0.558	-0.005 (-0.015 to 0.005)	0.306
TBLH lean mass				
MVPA	0.066 (-0.075 to 0.207)	0.354	**0.272 (0.091 to 0.454)**	**0.004**
Sport and exercise	0.202 (-0.215 to 0.620)	0.339	**0.343 (0.016 to 0.670)**	**0.040**
Sedentary time	-0.049 (-0.101 to 0.003)	0.064	0.021 (-0.059 to 0.102)	0.598
Screen time	0.110 (-0.031 to 0.252)	0.125	0.101 (-0.026 to 0.227)	0.118
Sleep	0.126 (-0.135 to 0.388)	0.339	-0.165 (-0.349 to 0.019)	0.077
**Males**				
TBLH BMC				
MVPA	0.003 (-0.005 to 0.011)	0.476	0.001 (-0.011 to 0.012)	0.887
Sport and exercise	0.012 (-0.000 to 0.025)	0.059	**0.027 (0.006 to 0.048)**	**0.012**
Sedentary time	-0.000 (-0.004 to 0.003)	0.966	-0.001 (-0.007 to 0.005)	0.700
Screen time	-0.002 (-0.007 to 0.004)	0.543	-0.001 (-0.008 to 0.007)	0.852
Sleep	-0.001 (-0.011 to 0.010)	0.918	-0.009 (-0.024 to 0.005)	0.215
TBLH lean mass				
MVPA	0.092 (-0.077 to 0.261)	0.282	0.164 (-0.059 to 0.387)	0.147
Sport and exercise	0.306 (-0.103 to 0.715)	0.141	**0.721 (0.327 to 1.115)**	**<0.001**
Sedentary time	0.016 (-0.068 to 0.099)	0.710	0.042 (-0.087 to 0.171)	0.522
Screen Time	0.063 (-0.056 to 0.181)	0.296	-0.091 (-0.222 to 0.039)	0.167
Sleep	**0.382 (0.138 to 0.626)**	**0.003**	-0.015 (-0.278 to 0.249)	0.913

*BMC* Bone mineral content, *MVPA* Moderate to vigorous physical activity, *TBLH* Total body less head

Values are unstandardised regression coefficients, their 95% confidence intervals, and *p*-values

The regression coefficient represents the difference in TBLH BMC and lean mass at age 15 to 17 years associated with a 10-minute average daily difference in each movement behaviour at age 6 to 9 years and age 9 to 11 years

Bold emphasis indicates statistical significance (*p* < 0.05)

^a^Estimates for TBLH BMC at age 15 to 17 years adjusted for age, stature, pubertal status, lean mass, fat mass and study group measured at age 15 to 17 years, and TBLH BMC measured at age 6 to 9 years. Estimates for TBLH lean mass at age 15 to 17 years adjusted for age, stature, pubertal status, fat mass and study group measured at age 15 to 17 years, and TBLH lean mass measured at age 6 to 9 years. MVPA and sedentary time are mutually adjusted for each other

^b^Estimates for TBLH BMC at age 15 to 17 years adjusted for age, stature, pubertal status, lean mass, fat mass and study group measured at age 15 to 17 years, and TBLH BMC measured at age 9 to 11 years. Estimates for TBLH lean mass at age 15 to 17 years adjusted for age, stature, pubertal status, fat mass and study group measured at age 15 to 17 years, and TBLH lean mass measured at age 9 to 11 years. MVPA and sedentary time are mutually adjusted for each other

At age 6 to 9 years to age 15 to 17 years, *n* = 78 females and 87 males for MVPA and sedentary time, *n* = 102 females and 112 males for sport and exercise and screen time, *n* = 79 females and 90 males for sleep

At age 9 to 11 years to age 15 to 17 years, *n* = 83 females and 75 males for MVPA and sedentary time, *n* = 103 females and 104 males for sport and exercise and screen time, *n* = 84 females and 76 males for sleep

**Fig. 2:**
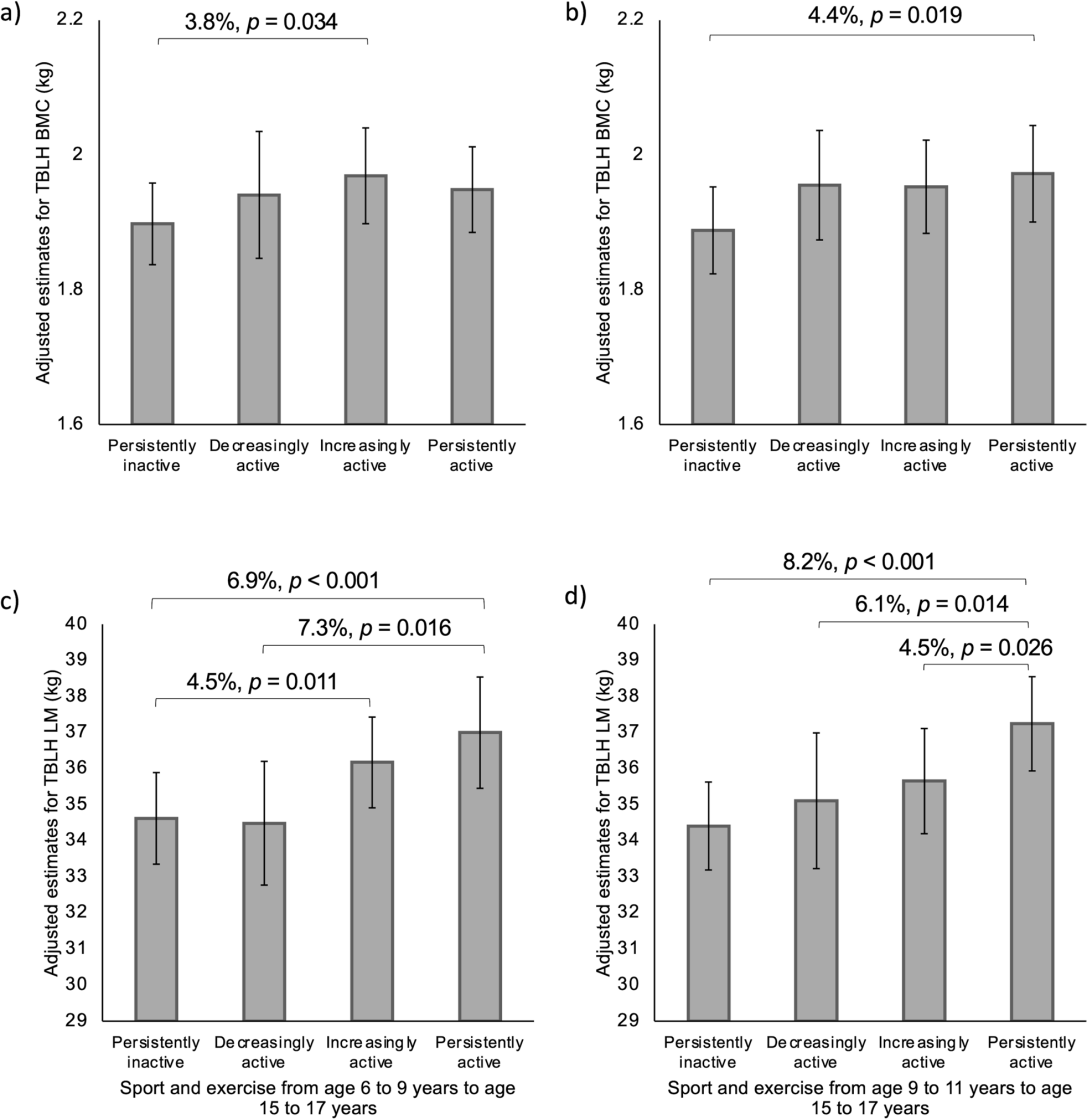
Differences in TBLH BMC and lean mass in females aged 15-17 years based on physical activity at age 6-9 years or age 9-11 years and age 15-17 years. Values are adjusted means (95% CI). Estimates for TBLH BMC are for a female at Tanner stage 4 in the control group with sex-specific mean levels of age, stature, lean mass and fat mass at measured at age 15 to 17 years, and TBLH BMC measured at age 6 to 9 years or age 9 to 11 years. Estimates for TBLH lean mass at age 15 to 17 years are for a female at Tanner stage 4 in the control group with sex-specific mean levels of age, stature, and fat mass at measured at age 15 to 17 years, and TBLH lean mass measured at age 6 to 9 years or age 9 to 11 years. a and c are for age 6 to 9 years to age 15 to 17 years. b and d are for age 9 to 11 years to age 15 to 17 years. For age 6 to 9 to age 15 to 17 years, the median split is based on 9 minutes at age 6 to 9 years and 15 minutes at age 15 to 17 years. For age 9 to 11 to age 15 to 17 years, the median split is based on 9 minutes at age 9 to 11 years and 14 minutes at age 15 to 17 years. For age 6 to 9 to age 15 to 17 years, *n* = 102 (persistently inactive *n* = 38, decreasingly active *n* = 13, increasingly active *n* = 26, persistently active *n* = 25). For age 9 to 11 to age 15 to 17 years, *n* = 103 (persistently inactive *n* = 36, decreasingly active *n* = 16, increasingly active *n* = 20, persistently active *n* = 31). BMC, bone mineral content; LM, lean mass; TBLH, total body less head

In terms of sport and exercise participation, from age 6 to 9 years to age 15 to 17 years, 38 females were persistently inactive, 13 were decreasingly active, 26 were increasingly active and 25 were persistently active. Increasingly active females from age 6 to 9 years to age 15 to 17 years had greater TBLH BMC at age 15 to 17 years than persistently inactive females (Fig. 2). Persistently active females from age 6 to 9 years to age 15 to 17 years had greater TBLH lean mass at age 15 to 17 years than persistently inactive and decreasingly active females, and increasingly active females had greater TBLH lean mass than persistently inactive females. From age 9 to 11 years to age 15 to 17 years, 36 females were persistently inactive, 16 were decreasingly active, 20 were increasingly active, and 31 were persistently active. Persistently active females from age 9 to 11 years to age 15 to 17 years had greater TBLH BMC at age 15 to 17 years than persistently inactive females. Persistently active females from age 9 to 11 years to age 15 to 17 years had greater TBLH lean mass at age 15 to 17 years than persistently inactive, decreasingly active, and  increasingly active females.

### Longitudinal associations of movement behaviours with TBLH BMC and lean mass in males

Sleep at age 6 to 9 years was positively associated with TBLH lean mass at age 15 to 17 years (Table 4). Sport and exercise participation at age 9 to 11 years was positively associated with TBLH BMC and lean mass at age 15 to 17 years (Table 4).

**Fig. 3:**
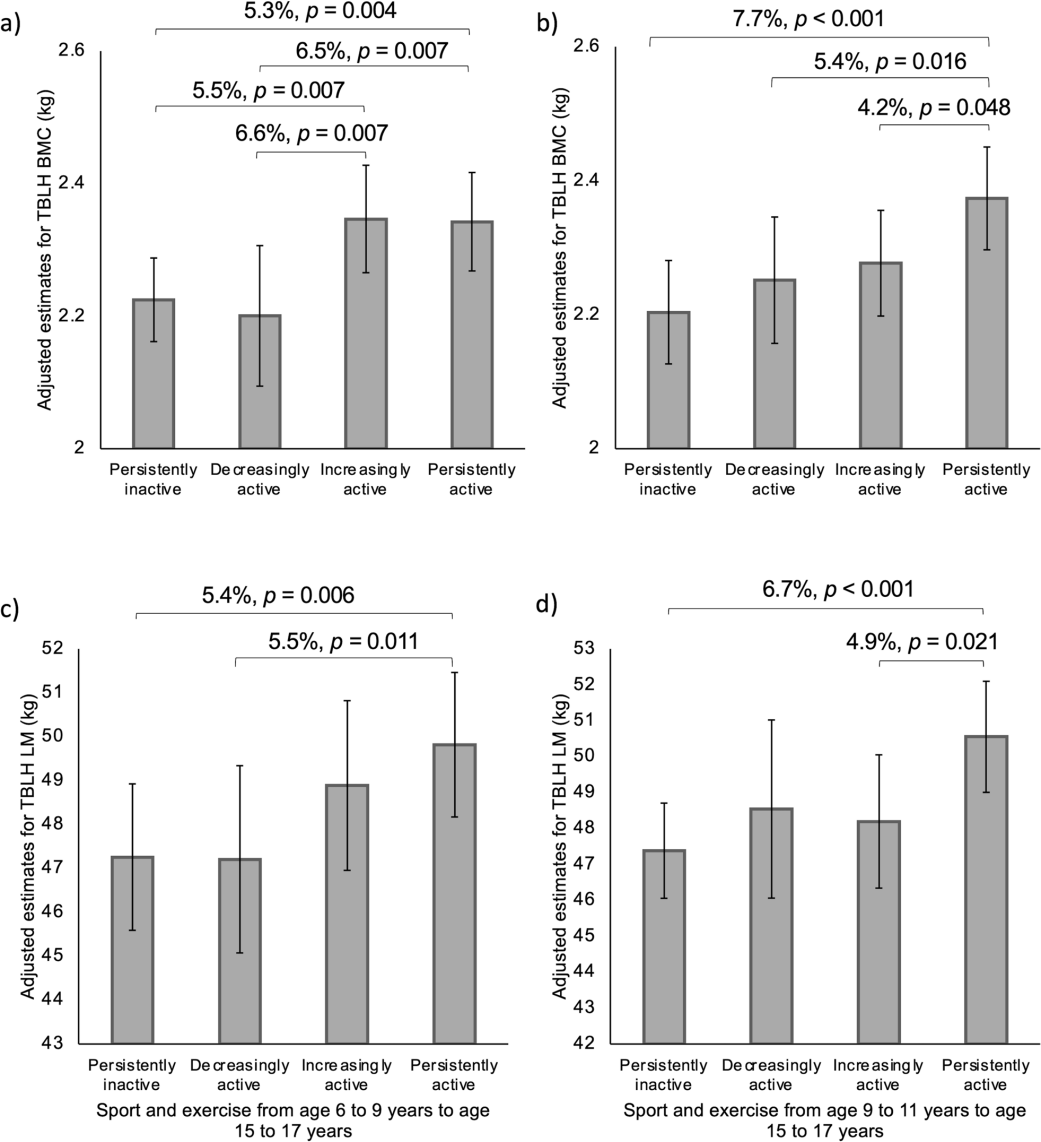
Differences in TBLH BMC and lean mass in males aged 15-17 years based on physical activity at age 6-9 years or age 9-11 years and age 15-17 years. Values are adjusted means (95% CI). Estimates for TBLH BMC are for a male at Tanner stage 4 in the control group with sex-specific mean levels of age, stature, lean mass and fat mass at measured at age 15 to 17 years, and TBLH BMC measured at age 6 to 9 years or age 9 to 11 years. Estimates for TBLH lean mass at age 15 to 17 years are for a male at Tanner stage 4 in the control group with sex-specific mean levels of age, stature, and fat mass at measured at age 15 to 17 years, and TBLH lean mass measured at age 6 to 9 years or age 9 to 11 years. a and c are for age 6 to 9 years to age 15 to 17 years. b and d are for age 9 to 11 years to age 15 to 17 years. For age 6 to 9 to age 15 to 17 years, the median split is based on 11 minutes at age 6 to 9 years and 18 minutes at age 15 to 17 years. For age 9 to 11 to age 15 to 17 years, the median split is based on 19 minutes at age 9 to 11 years and 19 minutes at age 15 to 17 years. For age 6 to 9 to age 15 to 17 years, *n* = 112 (persistently inactive *n* = 36, decreasingly active *n* = 20, increasingly active *n* = 20, persistently active *n* = 36). For age 9 to 11 to age 15 to 17 years, *n* = 104 (persistently inactive *n* = 37, decreasingly active *n* = 15, increasingly active *n* = 15, persistently active *n* = 37). BMC, bone mineral content; LM, lean mass; TBLH, total body less head

In terms of sport and exercise participation, from age 6 to 9 years to age 15 to 17 years, 36 males were persistently inactive, 20 were decreasingly active, 20 were increasingly active, and 36 were persistently active. Increasingly active and persistently active males from age 6 to 9 years to age 15 to 17 years had greater TBLH BMC at age 15 to 17 years than persistently inactive and decreasingly active males (Fig. 3). Persistently active males from age 6 to 9 years to age 15 to 17 years had greater TBLH lean mass at age 15 to 17 years than persistently inactive and decreasingly active males. From age 9 to 11 years to age 15 to 17 years, 37 males were persistently inactive, 15 were decreasingly active, 15 were increasingly active, and 37 were persistently active. Persistently active males from age 9 to 11 years to age 15 to 17 years had greater TBLH BMC at age 15 to 17 years than persistently inactive, decreasingly active and increasingly active males. Persistently active males from age 9 to 11 years to age 15 to 17 years had greater TBLH lean mass at age 15 to 17 years than persistently inactive and increasingly active males.

## Discussion

To the best of our knowledge, this study is the first to examine the cross-sectional and longitudinal associations of each of the 24-hour movement behaviours with TBLH BMC and lean mass in a general population from childhood to adolescence, including sport and exercise as a proxy measure of muscle and bone strengthening activity. Greater MVPA was associated with improved lean mass cross-sectionally in females and males, and with improved BMC and lean mass longitudinally in females. Higher levels of sport and exercise participation were associated with improved BMC and lean mass cross-sectionally and longitudinally in females and males, though it was important for children to maintain their activity levels into adolescence to maintain their improved TBLH BMC and lean mass. Sedentary time was inversely associated with lean mass in females and positively associated with lean mass in males, though these associations did not persist longitudinally. Screen time was negatively associated with BMC in males cross-sectionally but was not associated with BMC or lean mass longitudinally in females or males. Sleep was positively associated with BMC and inversely associated with lean mass in females cross-sectionally, and positively associated with lean mass in males longitudinally. Our findings highlight the importance of MVPA, sport and exercise participation, sedentary time, screen time and sleep for muscle and bone health.

The female and male participants of our study had similar median values of aBMD compared to reference values at age 6 to 9 years, lower median values at age 9 to 11 years, though these fell between the reference 10^th^ and 50^th^ percentile, and higher median values at age 15 to 17, though these fell between the reference 50^th^ and 90^th^ percentile, with cross-calibration equations applied to allow comparisons between different DXA systems [66, 67]. In terms of lean mass index, in order to account for differences in stature, the female and male participants had similar median values to reference values [68]. It is difficult to directly compare adherence to the 24-hour movement guidelines across studies due to the fact that that some studies have used self-reports and others devices to measure sleep and PA, and due to different epoch lengths, cut-points, wear sites, wear time protocols, and measurement periods used when measuring sleep and PA using devices [69]. Furthermore, the guidelines have been applied differently between studies. However, our sample appeared to have a higher adherence to the MVPA (66% to 85% in our sample compared to 44%), screen time (47% to 80% in our sample compared to 39%), and sleep guidelines (60% to 91% in our sample compared to 42%) at age 6 to 9 and age 9 to 11 years than in a 12-country study of children with mean age 10 years [70], though there are limited data in adolescents to compare to when the participants were age 15 to 17 years.

### Moderate-to-vigorous physical activity

The null cross-sectional associations of MVPA with TBLH BMC in both sexes that we observed are in contrast with the results of some previous studies, as outlined in a 2020 systematic review, which have found consistent positive cross-sectional associations of MVPA with whole-body DXA bone outcomes in males and in both sexes combined, but inconsistent associations in females [71]. Whereas we observed a longitudinal relationship between MVPA with TBLH BMC in females only, the systematic review found relatively consistent positive longitudinal associations between MVPA and whole body bone outcomes in females and males [71]. The differences between our findings and those of previous studies may be due to the different devices used to capture PA. In our study, a combined heart rate and movement sensor was used to estimate energy expenditure from acceleration and heart rate, providing a more accurate assessment of energy expenditure than acceleration alone, as was used in previous studies [45, 71]. Therefore, our measure of MVPA includes activities which increase heart rate with relatively small increases in acceleration, such as cycling, whereas previous studies have captured MVPA based purely on acceleration. As acceleration is more related to musculoskeletal loading than heart rate, previous studies may have been more likely to capture mechanically loaded MVPA only [72]. Given that mechanical loading is important for bone adaptation [73], this may have led to stronger associations between MVPA and bone outcomes in studies which have used acceleration-only estimates of MVPA compared to MVPA based on acceleration and heart rate, as used in our study, though this may also depend on how the accelerometry data is treated [74]. However, as the WHO PA guidelines do not stipulate that the recommended average 60 minutes daily MVPA should be mechanically loaded [12], previous studies may have overestimated the importance of MVPA for bone, as described in the guidelines. Our findings suggest that MVPA is largely not related to BMC in children and adolescents, though females who do more MVPA in childhood may have improved BMC in adolescence.

There is a lack of previous research investigating the associations of accelerometer-assessed MVPA with DXA-assessed lean mass. We observed inconsistent cross-sectional relationships between MVPA and lean mass in females and males, whereas previous studies have reported null associations in females and males [75, 76], or positive associations in females only, between MVPA and lean mass in children and adolescents [77]. Whilst we found that the association between MVPA and lean mass persisted longitudinally in females only, Zymbal and colleagues [14] observed that both females and males who accumulated more MVPA from age 5 to age 17 years had greater leg lean soft tissue at age 17 years [14]. It is unclear what accounts for the different observations across studies. As the longitudinal study by Zymbal and colleagues [14] used lower-limb measures of lean mass, it is plausible that the observed associations would be stronger than in other studies using whole body measures of lean mass, as the lower-limb is loaded during normal weight-bearing activity, and the accelerometer placement at the hip would be expected to be more sensitive to loading at the hip and lower-limb [78]. However, given the conflicting findings, further high-quality longitudinal studies are needed to extend our understanding of the relationship between MVPA and lean mass, though our findings indicate that concurrent MVPA may be important for lean mass in females and males, and that females who do more MVPA in childhood may have improved lean mass in adolescence.

### Muscle and bone strengthening activity

Although the positive cross-sectional associations of sport and exercise participation with TBLH BMC and lean mass were inconsistent across timepoints, we did find that sport and exercise participation at age 9 to 11 years was consistently positively associated with TBLH BMC and lean mass at age 15 to 17 years in females and males. Further, females and males who did more sports and exercise from childhood to adolescence had greater TBLH BMC and lean mass at age 15 to 17 years. Studies considering relationships between recreational sport and exercise participation with bone and lean mass in children and adolescents are scarce. In the Western Australia Pregnancy Cohort (Raine) Study, males who consistently participated in sport from age 5 to 17 years had greater whole-body BMC at age 20 than those who dropped out over time [79]. However, there were no differences in whole-body BMC in females based on their sport participation from age 5 to 17 years, and lean mass was not considered as an outcome [79]. The null association in females observed in the Raine cohort differs to our observations in females, potentially due to differences in measurement methods or covariates [79]. Whereas we considered average minutes per day of sport and exercise as our exposure in in both cross-sectional and longitudinal analyses, and categorised sport and exercise amounts into 4 groups as our exposure in further longitudinal analyses based on the average minutes per day from age 6 to 9 years to age 15 to 17 years, the Raine study used a binary indicator of whether the participants took part in any organised sport or not, which may contribute to the differences in findings [79]. A meta-analysis of exercise interventions, including jumping and/or circuit training, found a small, but statistically significant and beneficial effect of exercise on whole body BMC, further supporting our observations [80]. However, there was no effect of exercise interventions on lean mass [80]. It is possible that the activities captured within our measure of sport and exercise participation were more diverse than those used in intervention studies, offering a potential explanation for the relationship between sport and exercise participation and lean mass that we observed. Given the scarcity of studies investigating the relationships of sport and exercise participation with BMC and lean mass, further work is needed to better understand these relationships in general populations. Even so, our findings indicate that sport and exercise participation may be important for current BMC and lean mass, and that sport and exercise participation in childhood is important for BMC and lean mass in adolescence. Further, consistently participating in high levels sport and exercise across childhood and adolescence may lead to a greater BMC and lean mass at age 15 to 17 years. Given the current primary focus on aerobic activity in the WHO guidelines [12], our findings emphasise that the unique importance of muscle and bone strengthening activity for muscle and bone health should be promoted alongside MVPA, and included in future surveillance relating to the guidelines.

### Sedentary behaviour

The null cross-sectional and longitudinal associations of sedentary time with BMC that we observed is consistent with the conclusion of a systematic review of previous studies looking at accelerometer-measured sedentary behaviour with bone outcomes in children and adolescents [23]. However, whilst we found screen time was cross-sectionally negatively associated with TBLH BMC at age 15 to 17 years in males, the systematic review highlighted insufficient evidence for an associations of self- or parental-reported sedentary behaviour, which includes screen time, with total body bone outcomes [23]. In terms of lean mass, we observed inconsistent negative cross-sectional associations between sedentary time and lean mass in females, inconsistent positive cross-sectional associations between sedentary time and lean mass in males, and null longitudinal associations of sedentary time and screen time with lean mass. Previous studies have observed negative cross-sectional associations [25] and, similar to our findings, null longitudinal associations [24] between measures of sedentary behaviour and fat-free mass. The positive cross-sectional relationships between sedentary time and lean mass in males are unexpected, as there is no apparent biological reason why being sedentary would increase lean mass once fat mass is controlled for, as we did, though as the relationships do not persist longitudinally, it is possible that unmeasured confounding factors may be influencing the cross-sectional relationships. Although it is important to highlight that evidence supporting the inclusion of sedentary behaviour in the movement guidelines is largely based on adiposity, other cardiometabolic risk factors and psychosocial risk factors, our findings, combined with previous evidence, indicate that largely sedentary behaviour was not detrimental to BMC and lean mass [12, 23].

### Sleep

Sleep was largely unrelated to BMC in our study population, except for a positive association at age 15 to 17 years in females. Although there is limited research considering the associations between sleep and bone health, the largely null associations between sleep and TBLH BMC that we observed is consistent with previous research. In a population sample of European children and adolescents, adherence to the sleep guidelines, assessed with questionnaires, was not associated with bone stiffness index, as measured by quantitative ultrasound, though lean mass was not accounted for [28], and similar null findings have been observed in adults [27]. Taken together, our findings suggest that sleep duration is largely not related to BMC in children and adolescents, though it may be beneficial in adolescent females. In our sample, sleep was negatively cross-sectionally related to TBLH lean mass in females only at age 9 to 11 years, though this did not persist longitudinally. In males, sleep at age 6 to 9 years was positively associated with TBLH lean mass at age 15 to 17 years. Generally, previous studies have observed null associations between sleep duration and lean mass in children and adolescents [31, 81, 82]. However, in girls and boys age 10 to 12 years, sleep duration was positively associated with fat-free mass [25]. Differences in measures of sleep duration and lean mass may contribute to the different observations between studies. Whereas our study used DXA measures of lean mass, previous studies used DXA-assessed fat-free mass, which includes bone [31, 82], or estimated fat-free mass from skinfold measures [25, 81]. Further, some studies used device-measured sleep duration [31, 82], as did ours, whereas other studies used a self or parental report [25, 81]. Given the conflicting findings, further research would be valuable in better understanding the relationship between device-measured sleep duration and DXA-assessed lean mass, though our findings indicate that a longer sleep duration in childhood may contribute to a greater lean mass in adolescence in males.

### Clinical relevance

A 10-minute daily increase in MVPA at age 6 to 9 years was associated with 0.008 kg greater TBLH BMC at age 15 to 17 years in females, which equates to around 0.4 % of mean level of BMC at age 15 to 17 years. A 10-minute daily increase in MVPA at age 9 to 11 years was associated with 0.272 kg greater TBLH lean mass in females, which equates to around 0.8 % of the mean level of TBLH lean mass at age 15 to 17 years. A 10-minute daily increase in sport and exercise participation at age 9 to 11 years was associated with 0.020 kg greater TBLH BMC and 0.343 kg greater TBLH lean mass at age 15 to 17 years in females, which equates to around 1.0 % of the mean level of both BMC and lean mass at age 15 to 17 years. A 10-minute daily increase in sleep at age 6 to 9 years was associated with 0.382 kg greater TBLH lean mass at age 15 to 17 years in males, which equates to around 0.8 % of the mean level of lean mass at age 15 to 17 years. A 10-minute daily increase in sport and exercise at age 9 to 11 years was associated with 0.027 kg greater TBLH BMC and 0.721 greater TBLH lean mass at age 15 to 17 years in males, which equates to around 1.2 % of the mean level of BMC and 1.5 % of the mean level of lean mass at age 15 to 17 years. Given that theoretical modelling suggests a 10% increase in peak bone mass may delay the onset of osteoporosis by 13 years in later life [83], and that lean mass in key positive determinant of BMC [8], promoting MVPA, sport and exercise participation and sleep in childhood may contribute to delaying the onset of osteoporosis in later life.

### Strengths and limitations

The strengths of our study include the population-based sample of children with an 8-year follow-up into adolescence, device-measured MVPA, sedentary time and sleep, combined with questionnaire-assessed sport and exercise participation as a proxy for muscle and bone strengthening activity and screen time, and the measurement of BMC and lean mass by DXA. There are several limitations that should be considered when interpreting our findings. As we did not have a direct measure of muscle and bone strengthening activity, we were not able to directly assess the relationship between muscle and bone strengthening activity with BMC and lean mass. We used questionnaire-assessed participation in sport and organised exercise as a proxy measure for muscle and bone strengthening activity. Although previous studies assessing muscle and bone strengthening activities have measured participation in activities which would fall into the sport and organised exercise category, such as push-ups and weight-lifting, there may be other activities, such as swimming, which we have captured and would not be considered bone strengthening [84–87]. However, it has been reported that the most popular types of organised sports and exercise in Finland for boys are football (soccer), ice hockey, and floorball (a type of indoor hockey), and for girls are dance, gymnastics, aerobics, football (soccer), and horseback riding, all of which reflect muscle and bone strengthening activities [55, 88]. Even so, future studies with a direct assessment of muscle and bone strengthening activity would be valuable in better understanding the relationship between muscle and bone strengthening activity with BMC and lean mass in children and adolescents. The use of the 60-second epoch to capture MVPA may have led to an underestimation of MVPA, particularly when the participants were children [89], which may have influenced the observed relationships between MVPA with BMC and lean mass [90]. Further, we did not consider the relationships between LPA with BMC and lean mass in this study, so the associations between LPA with BMC and lean mass in children and adolescents remain unknown. Although DXA is the gold-standard for the assessment of BMC and lean mass in children, it is not possible to obtain true volumetric bone mineral density estimates from DXA, and as such the determinants of volumetric bone mineral density in our sample remain unknown. Further, as our sample had relatively high levels of MVPA at age 6 to 9 years and age 9 to 11 years, it is unknown whether these findings extend to less active children. Finally, although we adjusted for important covariates, residual confounding remains a potential limitation in all observational studies, and causality cannot be assumed.

## Conclusions

MVPA was largely not related to BMC in children and adolescents, though females who do more MVPA in childhood may have improved BMC in adolescence. Concurrent MVPA may be important for lean mass in females and males, and females who do more MVPA in childhood may have improved lean mass in adolescence. Sport and exercise participation may be important for current BMC and lean mass in females and males, and sport and exercise participation in childhood is important for BMC and lean mass in adolescence. Childhood sedentary behaviour was not detrimental to adolescent BMC and lean mass. Sleep duration was largely not related to BMC in children and adolescents, though it may be beneficial in adolescent females, and in males a longer sleep duration in childhood may contribute to a greater lean mass in adolescence. Overall, our findings suggest promoting engagement in the 24-hour movement behaviours in childhood, particularly sport and exercise to strengthen muscle and bone, is important in supporting bone and lean mass development in adolescence. Our findings support the inclusion and promotion of muscle and bone-specific activity in the physical activity guidelines. Given the particular importance of muscle and bone strengthening activity for bone and lean mass development, future research into the 24-hour movement behaviours should consider muscle and bone strengthening activity in addition to MVPA.

## Data Availability

The data that support the findings of this study are available from University of Eastern Finland but restrictions apply to the availability of these data, which were used under license for the current study, and so are not publicly available. Data are however available from the authors upon reasonable request and with permission of University of Eastern Finland and PANIC study Principal Investigator (timo.lakka@uef.fi).

## Abbreviations

aBMD: Areal bone mineral density
BMC: Bone mineral content
BMI: Body mass index
DXA: Dual energy X-ray absorptiometry
IOTF: International Obesity Task Force
IQR: Interquartile range
LPA: Light physical activity
MVPA: Moderate-to-vigorous physical activity
PA: Physical activity
PANIC: Physical Activity and Nutrition in Children
SD: Standard deviation
SDS: Standard deviation score
TBLH: Total body less head
WHO: World Health Organisation
